# Rapid Tuberculosis Diagnosis Using Reporter Enzyme Fluorescence

**DOI:** 10.1128/JCM.01462-19

**Published:** 2019-11-22

**Authors:** Preeti Sule, Ronak Tilvawala, Toriq Mustapha, Hany Hassounah, Aneesa Noormohamed, Suprateek Kundu, Edward A. Graviss, Grant K. Walkup, Ying Kong, Jeffrey D. Cirillo

**Affiliations:** a Department of Microbial Pathogenesis and Immunology, Texas A&M Health Science Center, Bryan, Texas, USA; b Department of Biostatistics & Bioinformatics, Emory University, Atlanta, Georgia, USA; c Department of Pathology and Genomic Medicine, Houston Methodist Research Institute, Houston, Texas, USA; d Agios Pharmaceuticals, Cambridge, Massachusetts, USA; e Department of Microbiology, Immunology and Biochemistry, University of Tennessee Health Science Center, Memphis, Tennessee, USA; Carter BloodCare & Baylor University Medical Center

**Keywords:** *Mycobacterium tuberculosis*, biomarker, point of care, BlaC, lactamase

## Abstract

Tuberculosis is the most frequent cause of death in humans from a single infectious agent. Due to low numbers of bacteria present in sputum during early infection, diagnosis does not usually occur until >3 to 4 months after symptoms develop. We created a new more sensitive diagnostic that can be carried out in 10 min with no processing or technical expertise. This assay utilizes the Mycobacterium tuberculosis-specific biomarker BlaC in reporter enzyme fluorescence (REF) that has been optimized for clinical samples, designated REFtb, along with a more specific fluorogenic substrate, CDG-3. We report the first evaluation of clinical specimens with REFtb assays in comparison to the gold standards for tuberculosis diagnosis, culture and smear microscopy. REFtb assays allowed diagnosis of 160 patients from 16 different countries with a sensitivity of 89% for smear-positive, culture-positive samples and 88% for smear-negative, culture-positive samples with a specificity of 82%. The negative predictive value of REFtb for tuberculosis infection is 93%, and the positive predictive value is 79%. Overall, these data point toward the need for larger accuracy studies by third parties using a commercially available REFtb kit to determine whether incorporation of REFtb into the clinical toolbox for suspected tuberculosis patients would improve case identification. If results similar to our own can be obtained by all diagnostic laboratories, REFtb would allow proper treatment of more than 85% of patients that would be missed during their initial visit to a clinic using current diagnostic strategies, reducing the potential for further spread of disease.

## Abstract (precis)

Tuberculosis is the most frequent cause of death in humans from a single infectious agent. Due to low numbers of bacteria present in sputum during early infection, diagnosis does not usually occur until >3 to 4 months after symptoms develop. We created a new more sensitive diagnostic that can be carried out in 10 min with no processing or technical expertise.

## INTRODUCTION

In 2014, the World Health Organization (WHO) made a projected target of reducing the tuberculosis (TB) burden 90% by 2035, which would indicate cessation of the ongoing global epidemic (1). Rapid, sensitive, and low-cost diagnostics for early stages of patient care or point of care (POC) are an urgent need in the fight against tuberculosis. Development of POC diagnostics would be facilitated by identification of new biomarkers and simplification of testing platforms, allowing a new test to be effectively used in resource limited environments (2). Progress in tuberculosis-associated biomarker discovery has been slow, and implementation of biomarkers for diagnostics has been challenging (3–8). Recent advances have led to the development of several effective diagnostics, including GeneXpert MTB/RIF (9), with the ability to detect Mycobacterium tuberculosis and test for drug resistance in less than 2 h (3, 10). Despite several attempts to have GeneXpert more widely utilized, the cost associated with the assay has been a roadblock to utilization in all areas (10–12). The gold standard tests, culture and smear microscopy, are lab based and usually require multiple visits to the clinic before conclusive diagnosis (12–15), often taking weeks to months before results are obtained and frequently becoming contaminated, preventing definitive results (16–19). Smear microscopy is usually the first test administered, but the low sensitivity often allows infection to be transmitted to others before diagnosis (1, 16, 20). In the absence of appropriate treatment, the mortality rate for tuberculosis can be as high as 50%, creating a grim scenario in resource-limited countries (21). Unfortunately, progress in the development of new diagnostics has been extremely slow (22). Delayed diagnosis increases morbidity and mortality, prevents control of drug resistance, and results in inefficient use of monetary resources and manpower (22).

**FIG 1:**
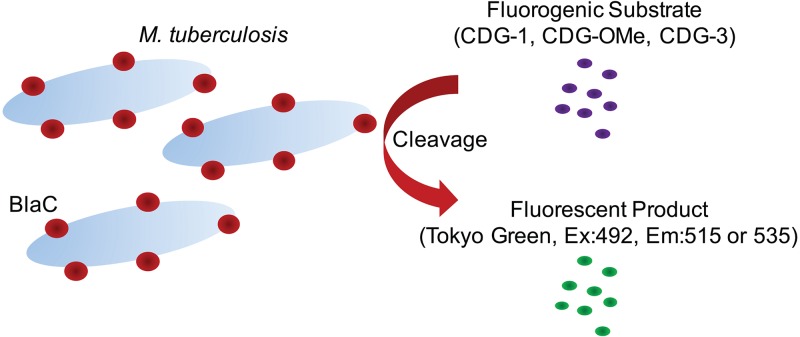
Illustration of the reporter enzyme fluorescence (REF) assay used to evaluate clinical specimens. The M. tuberculosis β-lactamase, BlaC, is surface localized (∼90%) and secreted (∼10%) when the bacteria are grown under laboratory conditions in liquid medium (28), making it easily accessible to fluorogenic substrates for REF. The fluorogenic substrates CDG-1, CDG-OMe, and CDG-3 produce the fluorescent product Tokyo green after cleavage by BlaC. The catalytic activity of BlaC can be measured by detecting the presence of fluorescence using a plate reader or similar system that allows excitation at 492 nm and fluorescence emission at 515 or 535 nm. Although a 515-nm emission was used for all *in vitro* studies due to the maximal emission of Tokyo green at this wavelength, 535 nm was chosen for use with clinical specimens because this wavelength displays a lower autofluorescent background due to components of clinical sputum samples.

Imaging technologies using radiological and optical probes have recently shown the potential to instantaneously quantify bacteria and other infectious agents, offering a viable alternative to culture (23–35). Unfortunately, most approaches for detection and quantification of bacteria require the use of recombinant strains expressing fluorescent or bioluminescent reporters (23–27, 30, 35–38), preventing application to patients because clinical strains do not express these reporters. Use of endogenous enzymes in pathogens to produce signals for imaging using custom probes shows promise for detection and quantification of clinical strains (28, 39–41), and their catalytic nature offers the potential for extremely high sensitivity (42). In the case of tuberculosis, the constitutively expressed (43, 44), surface localized and secreted (28), robustly catalytic enzyme BlaC (Rv2068c) (45, 46) is extremely sensitive for detection of M. tuberculosis using reporter enzyme fluorescence (REF) (28, 40). Although early fluorogenic substrates did not have specificity for BlaC over other β-lactamases, the unique active site of the BlaC (47) makes synthesis of highly specific substrates feasible. Custom fluorogenic substrates that display high specificity for BlaC over other β-lactamases have been synthesized but have not been well validated in clinical samples (48–50). The specificity of these substrates combined with the extremely high catalytic rate of BlaC can allow rapid detection of 1 to 10 M. tuberculosis bacilli within sputum (Fig. 1), even when numerous other species of bacteria expressing β-lactamase are present (48–50), suggesting that these specific substrates will perform well in clinical samples. Since the development of a low-cost, sputum-based triage test is an immediate need for tuberculosis control programs (51), it has been suggested that a biomarker assay like the BlaC-based REF assay using BlaC, designated REFtb, could be employed at the POC and greatly increase tuberculosis case identification and, thereby, appropriate treatment (52).

In the current study, we completed development of the REFtb diagnostic system and report the first diagnostic trial comparing REFtb with the gold standards of culture and smear microscopy. We evaluated conservation of BlaC in tuberculosis complex bacteria and compared it to β-lactamases present in other bacterial species. We synthesized the BlaC-specific REF substrate CDG-3 and found that it has high selectivity for M. tuberculosis BlaC and can quantitatively measure enzyme and bacterial levels in sputum. These characteristics allowed development of a simple sputum-based diagnostic assay for tuberculosis that can now be evaluated in patients (48–50). In the current study, we optimized conditions for REFtb to diagnose tuberculosis using sputum and conducted the first blinded trial of REFtb in patients with clinical symptoms of tuberculosis. We found that, based on the sensitivity and specificity obtained using BlaC-based REF for diagnosis of tuberculosis, REFtb represents a promising new triage test for tuberculosis that could be used to improve case identification prior to follow up by appropriate treatment and confirmation with other diagnostic strategies.

## MATERIALS AND METHODS

### Diagnostic process.

The sample collection and processing, including the rendering of the diagnostic assay was carried out in a blinded manner, wherein the personnel responsible for doing the experiments were not aware of the acid-fast stain (smear) or culture status of the patients providing samples. The clinical samples were provided by The Houston Methodist Research Institute (Houston, TX) and the Foundation for Innovative New Diagnostics (FIND, Geneva, Switzerland). Initial sample volume was variable from 100 μl to 1 ml, with the majority of samples having a >500-μl initial volume. Samples from Houston were consecutive and taken over 4 months, initially selected as likely true tuberculosis-positive patient samples. Samples from FIND were selected to have an equal distribution of negative, culture-positive, and both smear- and culture-positive sample type, with one-third of each type. Samples were randomized and coded prior to shipping with no personnel involved in testing able to decode the sample type. The samples were processed for smear and culture at primary care centers, coded, and frozen in aliquots. These samples were transported over dry ice to Texas A&M where they were stored at −80°C until use. Clinical samples were thawed at room temperature (RT) before further processing. The REFtb assay was carried out as reported earlier (49). Briefly, samples were thawed to room temperature, and an equal volume of transport stabilization solution (TSS) was added. The samples were incubated in TSS for 1 h before reporter enzyme fluorescence solution (49) containing CDG-3 to give a final concentration of 1 μM (REFS) was added. Cleaved CDG-3 was read using a 490-nm excitation and an emission of 515 nm or 535 nm. Optimal specificity and sensitivity were obtained with clinical samples using 535 nm, and all data presented utilize this emission wavelength. The samples were then read by a 100-point scan in a 10 by 10 grid pattern using a spectrometer (Mithras LB 940; Berthold Technologies, Oakridge, TN) immediately and after 10 min incubation at room temperature (25°C). Each set of assays also evaluated a TSS control comprised of TSS with tuberculosis-negative sputum.

### Statistical analysis.

We determined the cutoff for clinical samples that yielded optimal sensitivity and specificity. Two methods were used to determine cutoffs with SAS version 9.4 with the ROC macro as follows: (i) determination of a threshold for the entire data set that was then used to call samples and (ii) batch-specific thresholding, where cutoffs were determined for each batch of samples prior to making calls for that batch. Once we determined the appropriate cutoff for optimal sensitivity and specificity, this cutoff was used for analysis of all data and compared to standard diagnostic tests. This comparison was made for smear-positive and culture-positive samples and smear-negative and culture-positive samples. Sensitivity was calculated for the REFtb assay against both groups of samples, either smear negative and culture positive or both smear and culture positive. Specificity was calculated against samples that were both smear and culture negative. Clinical samples were called positive if their fluorescence was higher than the stated cutoff and negative if their fluorescence was lower. We determine the cutoffs for the k^th^ sample as T × c_k, where c_k is the fluorescence for the control in the k^th^ sample and T is a multiplier determined to yield maximum sensitivity and specificity. Sensitivity is defined as the ratio of the true positives to the total number of positives and is related to the power of a test, whereas specificity is defined as the ratio of true negatives to the total number of negatives and can be interpreted as one minus the false discovery rate. The true positives, false positives, true negatives, and false negatives were determined using standard suspected tuberculosis clinical sample culture and acid-fast stain microscopy to classify the sputum samples. Where appropriate, a 95% confidence interval is presented for data obtained. Biological experiments were repeated at least twice in triplicate, and data presented are means and standard deviations. Significant differences were determined using analysis of variance (ANOVA) with the Tukey-Kramer *post hoc* pairwise *t* test for multiple comparisons and the Student's *t* test for individual pairwise comparisons. *P* values of less than 0.05 were considered significant.

### Bioinformatic analyses.

Conservation of the mycobacterial β-lactamase BlaC within the tuberculosis complex was examined by obtaining protein sequences from NCBI and aligning them using ClustalW (53). The signature motifs for class A β-lactamases were selected within BlaC based on previous publications (47, 54). The evolutionary relatedness between BlaC and β-lactamases from other bacterial species was examined by obtaining sequences from the NCBI protein database (55, 56) and aligning them using Clustal Omega version 1.2.4 (57). The amino acid sequence of BlaC from 60 to 170, spanning the active site residues, was selected for comparison with the same region from other β-lactamases. The aligned sequences were edited to include the amino- and carboxy-terminal sequences. Protein sequences of β-lactamases were aligned and then a phylogenetic tree was constructed (58). A simple unrooted tree depicting relatedness of β-lactamases was generated.

### Bacterial strains and growth conditions.

Mycobacterium tuberculosis subsp. *bovis* bacillus Calmette-Guérin (BCG) carries BlaC and produces similar levels of BlaC as other M. tuberculosis strains (28). BCG was cultured as described previously (40) in 7H9 broth (Difco, Detroit, MI) supplemented with 0.5% glycerol, 10% oleic acid dextrose complex without catalase (OADC), and 0.05% Tween 80. Bacterial cultures were incubated at 37°C in the presence of 5% CO_2_ until an optical density at 600 nm of 0.5. The bacterial culture was centrifuged and washed with 7H9 liquid medium without any supplements and resuspended in this same medium prior to use.

### Fluorogenic probes.

The fluorogenic probes CDG-1, CDG-OMe, and CDG-3 were synthesized and validated as described previously (48, 49). Probes were stored at −80°C in dimethyl sulfoxide (DMSO) until use.

### Purification of BlaC and other β-lactamases.

Expression and purification of BlaC and TEM-1 were carried out as described previously (47, 49). Expression and purification of AmpC, P99, KPC-2, and CTX-M-15 β-lactamases were carried out and provided by AstraZeneca (Waltham, MA). Activity of purified enzymes was validated and quantified by nitrocefin assays as described previously (59). Briefly, 0.2 nM of each enzyme in 2-(*N*-morpholino)ethanesulfonic acid (MES), pH 6.0, was incubated with 200 μM nitrocefin at room temperature (25°C) to estimate active enzyme units. All activity assays were carried out in 100 μl final volume. Activity of all enzymes used was determined the same day or within a few days of use. No loss of enzyme activity has been observed under standard storage conditions (47).

### β-Lactamase activity assays.

Equal units, as determined by nitrocefin activity, of each β-lactamase were used to compare the specificity of CDG-3 with CDG-1 and CDG-OMe. Two β-lactamases, TEM-1 (class A β-lactamase) and AmpC (class B β-lactamase) were examined using various concentrations of enzyme up to 500 nM. All enzyme assays were conducted at room temperature (25°C). Briefly, 0.1 μM fluorogenic substrate was incubated with different concentrations of β-lactamase for various periods of time in the dark. Enzyme and substrate dilutions were prepared in MES buffer (pH 6.0), and the assay was carried out in 96-well plates using an EnVision plate reader (PerkinElmer, Waltham, MA). Emission spectra upon excitation at 490 nm were collected and compared with and without BlaC. Standard nitrocefin assays (59) with various concentrations of BlaC were first carried out to standardize enzyme concentrations, and then dilutions were made and activity was determined in sputum, sputum with 200 mM MES buffer, pH 6.0, plus 2% dithiothreitol (DTT), designated TSS, and MES buffer using each of the fluorogenic substrates.

### Detection of BlaC in sputum.

Tuberculosis-negative human sputum was obtained from Robert Fader at Baylor Scott & White (Temple, TX). Sputum samples were stored at −80°C, thawed at room temperature (25°C), and mixed with an equal volume of TSS. β-Lactamases diluted in MES buffer were added (final concentrations of 100 to 500 nM) and incubated for 1 h. After 1 h of incubation, fluorogenic substrates in REF solution (49) were added to each sample (final concentration of 1 μM). The subsequent change in fluorescence of the solution was monitored spectrophotometrically with excitation at 490 nm and emission at 515 nm. All of the assays, unless noted otherwise, were carried out in 96-well plates in triplicate.

### Detection of bacteria in sputum.

BCG was used as a control for the ability to detect M. tuberculosis in sputum as described previously (49). After estimating bacterial numbers by optical density at 600 nm, ∼10^7^ CFU of BCG were centrifuged, the supernatant removed and resuspended in the same medium. Dilutions were made, and ∼100 CFU were added to pooled tuberculosis-negative human sputum. Samples were diluted 1:1 in TSS and incubated for 1 h at room temperature (25°C). Substrate concentrations varied from 0.01 μM to 10 μM in 200 mM MES, and samples were read immediately after adding the substrate and every 20 min for 3 h at room temperature in 24-well plates using a Mithras LB 940 plate reader (Berthold Technologies, Oakridge, Tennessee) at 490 nm excitation and 515 nm emission. Tuberculosis-negative sputum without BCG was included as a negative control in all experiments.

## RESULTS

### M. tuberculosis BlaC is an unusual β-lactamase.

**FIG 2:**
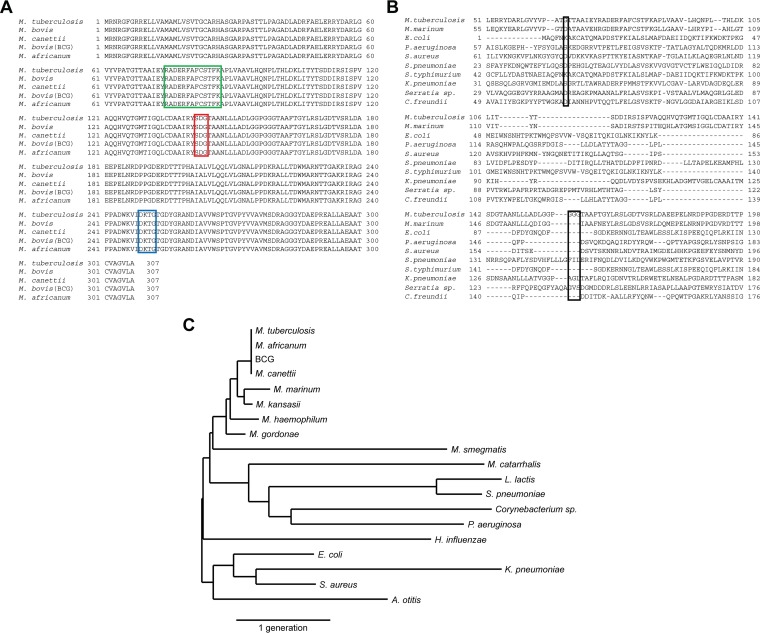
Characteristics of M. tuberculosis BlaC that make it broadly applicable as a biomarker to detect infection. (A) Amino acid sequence alignment of the M. tuberculosis BlaC with the β-lactamases present in tuberculosis complex bacteria showing that the protein sequence is completely conserved in all bacteria that can cause tuberculosis in humans and Mycobacterium tuberculosis subsp. *bovis* bacillus Calmette-Guérin (BCG). Motifs I, II, and III that are present in class A β-lactamases are marked by green, red, and blue boxes, respectively, on the alignment (47, 54). (B) Amino acid sequence alignment with a diverse set of other β-lactamases. Boxed amino acids within this alignment indicate unique glycine residues found in the amino acid sequence for BlaC. (C) Phylogenetic tree illustrating the evolutionary distances between M. tuberculosis BlaC and other selected β-lactamases. The scale bar denotes the distance equivalent to 0.8 generations for the phylogenetic tree.

The BlaC β-lactamase produced by M. tuberculosis is a biomarker for the presence of viable bacteria during infections in humans and animals (28, 40, 49, 50). This enzyme is secreted through a twin-arginine translocation (Tat) pathway (60, 61), resulting in BlaC being primarily cell associated and membrane localized (28). β-Lactamase activity in M. tuberculosis strains is almost exclusively due to BlaC, since mutations in the *blaC* gene lead to production of negligible β-lactamase activity by the resulting strains (28, 61). Based on examination of microarray data in the Gene Expression Omnibus (GEO) at the National Center for Biotechnology Information (NCBI) and ArrayExpress at the European Bioinformatics Institute (EBI) databases, *blaC* expression is constitutive and not influenced by growth conditions. During infections, there are no significant differences between the first 24 h postinfection (62), under aerobic conditions (63), or during an oxidative stress response (64). Comparison of BlaC with tuberculosis complex members, those mycobacteria that cause tuberculosis, finds conservation throughout the protein (Fig. 2A). BlaC is a class A β-lactamase since it carries the three motifs characteristic of this enzyme class (47, 54). Class A β-lactamases from other bacteria have little similarity to BlaC outside of these motifs (Fig. 2B). Interestingly, the glycine near motif I and the three glycines near motif II are not present in other β-lactamases, and they map to the binding pocket in the crystal structure (47, 48). These unusual glycines within the BlaC active site are what make the M. tuberculosis β-lactamase unique. BlaC is not found in other mycobacterial species or any bacterial species commonly found in the human oropharynx (65) (Fig. 2C). Overall, these observations suggest BlaC is specific to tuberculosis complex members, even within mycobacterial species.

### CDG-3 is a highly specific and sensitive fluorogenic probe for M. tuberculosis.

**FIG 3:**
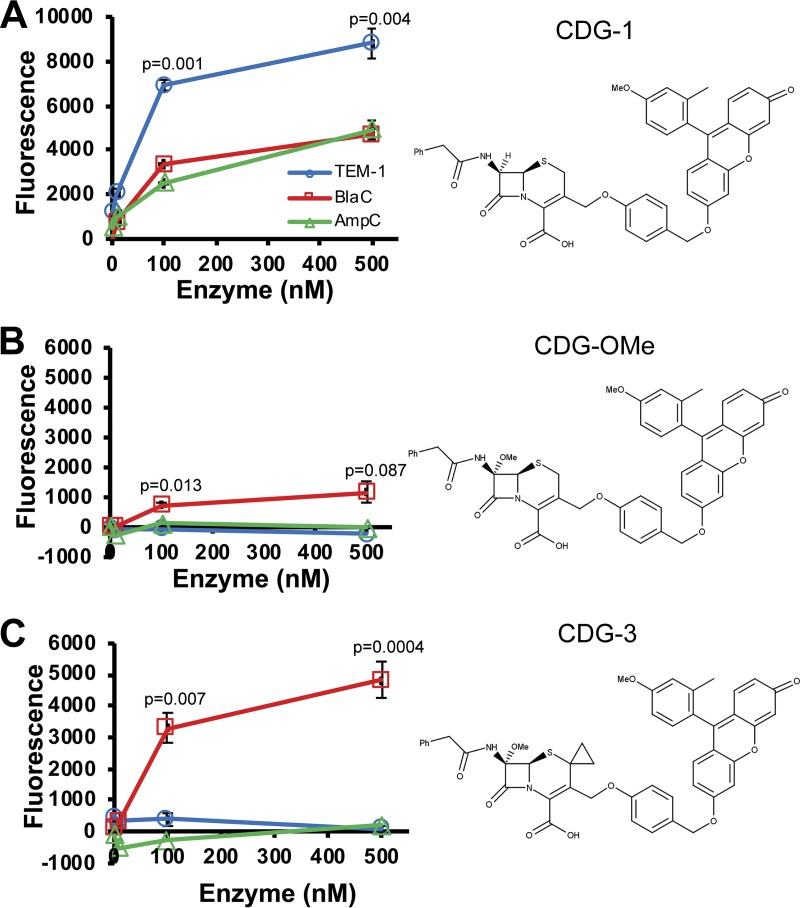
Fluorescence (excitation, 490 nm; emission, 515 nm) obtained using different fluorogenic probes for reporter enzyme fluorescence (REF) with the TEM-1, AmpC, and BlaC β-lactamases. Fluorescence after 3 h coincubation of 1 μM CDG-1 (A), CDG-OMe (B), and CDG-3 (C) with between 0.1 and 500 nM each β-lactamase at room temperature in MES buffer, pH 6.0. Data points shown represent the average fluorescence from samples done in triplicate, and error bars represent the standard deviations for these data. *P* values are shown above data points and are derived from comparison of data for BlaC with that of TEM-1 by ANOVA. Structure for the probe used is shown to the right of their graph for activity with the β-lactamases.

We designed three fluorogenic substrates that can sensitively detect BlaC, CDG-1, CDG-OMe, and CDG-3 (48–50). The specificity of these substrates is important because of the need for high specificity in patient samples where many other β-lactamases are likely present (48). Although specificity of CDG-OMe compared to TEM-1 (class A) β-lactamase has been examined (48), these substrates have not been directly compared for temporal kinetics to each other or with other β-lactamases. When we compared all three substrates directly, we found that the cephalosporin-based fluorogenic substrate CDG-1 is cleaved more readily by TEM-1 (class A) than by BlaC and AmpC (class C) β-lactamases (Fig. 3A). CDG-OMe, with a methoxy substitution at the 7-amino position, is cleaved more readily by BlaC than TEM-1 and AmpC (Fig. 3B). Selectivity of CDG-OMe for BlaC over TEM-1 is much better than that over AmpC (Table 1). CDG-3, with both a methoxy substitution at the 7-amino position and a cyclopropyl substitution at the 2 position, displays greater selectivity for BlaC than CDG-OMe (Fig. 3C). We found that CDG-3 displays high specificity for BlaC over other β-lactamases, even KPC-2, which is thought to be very versatile (66, 67). Based on these data, we examined the use of CDG-3 to detect M. tuberculosis infection.

**TABLE 1:** Specificity of substrates for β-lactamases

β-lactamase	CDG-OMe activity (%)^*a*^	CDG-3 activity (%)^*b*^
BlaC^*c*^	100 ± 9.29	100 ± 2.00
TEM-1^*d*^	5.74 ± 0.55	3.44 ± 0.08
AmpC^*d*^	26.7 ± 2.66	6.82 ± 0.28
KPC-2^*d*^	53.3 ± 3.28	17.9 ± 1.07
CTX-M-15^*e*^	5.91 ± 0.53	2.79 ± 0.39
P99^*d*^	7.22 ± 0.75	2.15 ± 0.13

a Activity was calculated as the percentage of fluorescence (excitation, 490 nm; emission, 515 nm) after coincubation of 0.2 nM of each β-lactamase with 1 μM CDG-OMe for 40 min at room temperature (25°C).

b Activity was calculated in exactly the same manner as for CDG-OMe, except that 1 μM CDG-3 was used instead of CDG-OMe.

c *P* < 0.0001 for BlaC compared to all other β-lactamases for both CDG-OMe and CDG-3.

d *P* < 0.01 for CDG-3 compared to CDG-OMe for this β-lactamase.

e *P* < 0.05 for CDG-3 compared to CDG-OMe for this β-lactamase.

### Detection of BlaC enzyme in clinical material.

**FIG 4:**
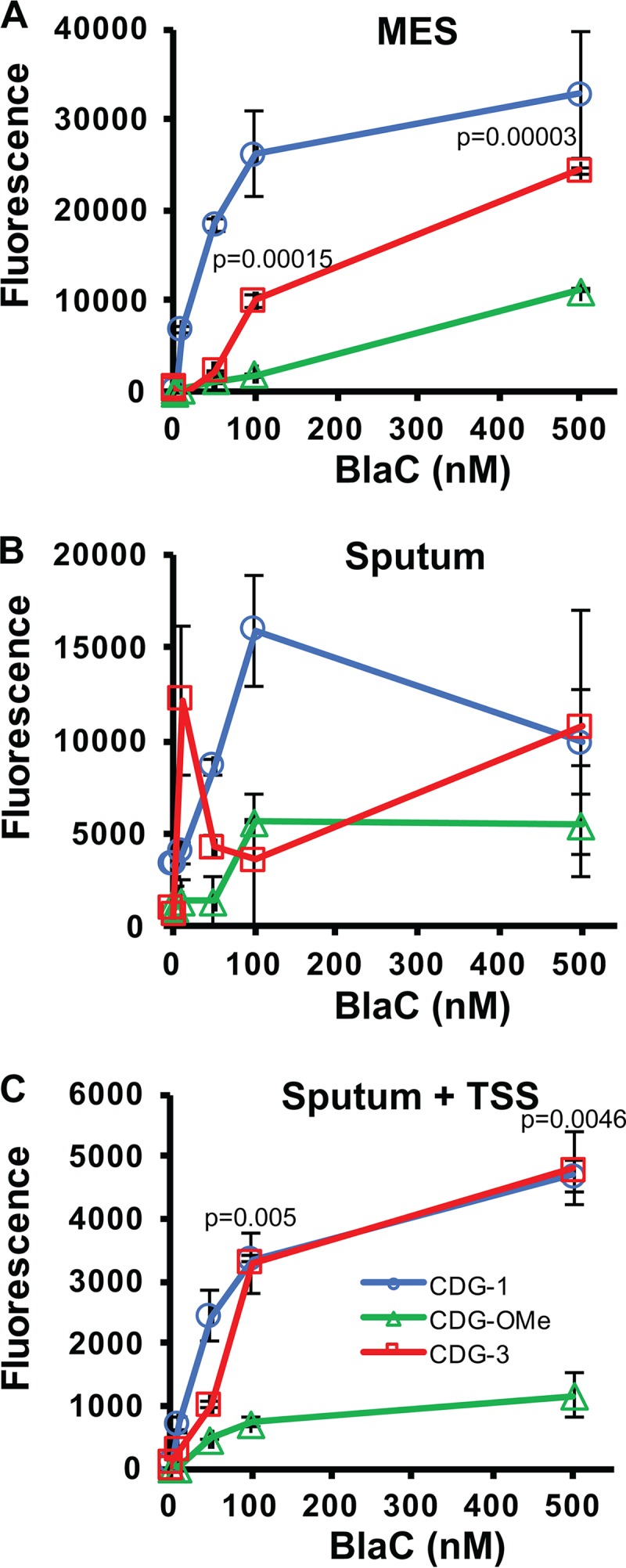
Detection of different concentrations of BlaC (0.1 to 500 nM) with fluorogenic probes CDG-1, CDG-OMe, and CDG-3 at 1 μM in MES buffer, pH 6.0 (A); sputum alone (B); and sputum with transport stabilization solution (TSS) (C) at room temperature (25°C) for 3 h. Data points shown represent the average fluorescence from samples done in triplicate, and error bars represent the standard deviations for these data. *P* values are shown above data points and are derived from comparison of data for CDG-3 with that of CDG-OMe by ANOVA.

**FIG 5:**
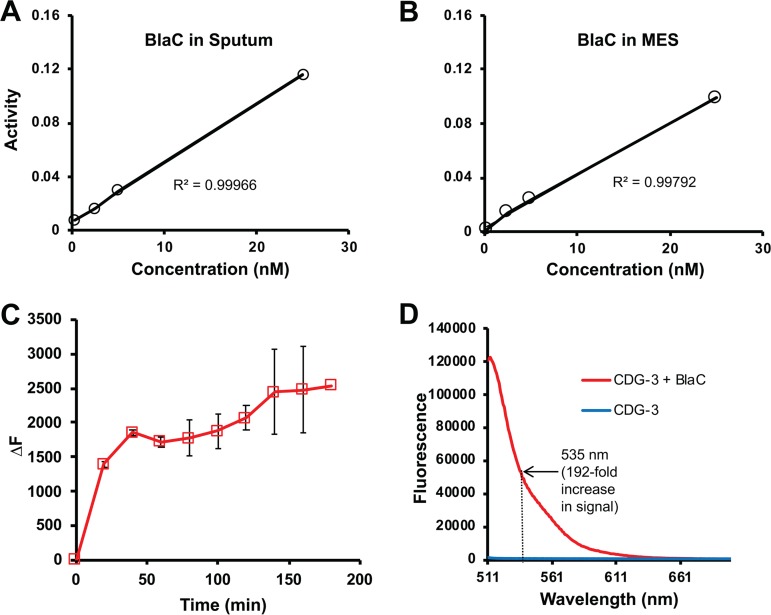
Reporter enzyme fluorescence probes behave similarly in buffer and sputum containing transport stabilization solution (TSS). Correlation of activity (change in fluorescence per minute, excitation, 490 nm; emission, 515 nm) from 1 μM CDG-3 incubated with different concentrations of BlaC in sputum containing TSS (A) and MES buffer, pH 6.0, (B) for 40 min at room temperature (25°C). Linear correlation value (*R*^2^) is shown on both graphs. (C) Change in fluorescence for 1 μM CDG-3 in the presence of 0.2 nM BlaC in sputum over time at room temperature (25°C). Change in fluorescence (ΔF) is calculated as the fluorescent signal at each time point minus the signal immediately after adding the substrate. All experiments were carried out in duplicate, and the averages and standard deviations reported. (D) Fluorescence emission spectrum of CDG-3 before and after treatment with 0.2 nM BlaC for 40 min in sputum at room temperature (25°C) with excitation at 490 nm.

The primary clinical material used for diagnosis of tuberculosis is sputum used for acid-fast staining and culture (1, 22, 52, 68). We assessed the ability of the fluorogenic substrates to detect purified BlaC spiked into tuberculosis-negative human sputum. CDG-1, CDG-OMe, and CDG-3 allow sensitive detection of BlaC, and the signal correlates well with concentration of enzyme (Fig. 4A). Interestingly, human sputum interferes with detection of BlaC (Fig. 4B). We found that stabilization of pH with MES buffer and inclusion of DTT, designated TSS, allows quantitative detection of BlaC in sputum (Fig. 4C). In the case of sputum with TSS, CDG-1 and CDG-3 generated a greater signal from BlaC than CDG-OMe. We found that there was a very strong correlation (*r*^2^ = 0.99966; *P* = 0.016) between BlaC concentration in sputum and fluorescence (Fig. 5). The correlation of fluorescence with the concentration of BlaC is similar in sputum and MES buffer (*r*^2^ = 0.99792; *P* = 0.041). The reaction of BlaC with CDG-3 occurs rapidly in sputum, generating a nearly maximal fluorescent signal within the first 20 min (Fig. 5C). We found that, similar to cleavage of CDG-3 by BlaC in buffer alone (49), CDG-3 in sputum with TSS gives a 238- and 192-fold increase in fluorescence at 515 and 535 nm emission, respectively (Fig. 5D). We were interested in using 535 nm, even though similar data would be obtained *in vitro* at both wavelengths, since examination of clinical material by ourselves and other investigators suggests that collecting emission at longer wavelengths helps to reduce interference from hemoglobin that may sometimes be present in sputum (40, 49, 69). These data suggest that CDG-3 can be used to accurately detect M. tuberculosis BlaC in sputum, making it an excellent candidate for development of an REF diagnostic assay for tuberculosis.

### REF assays can detect 100 CFU of M. tuberculosis in sputum.

**FIG 6:**
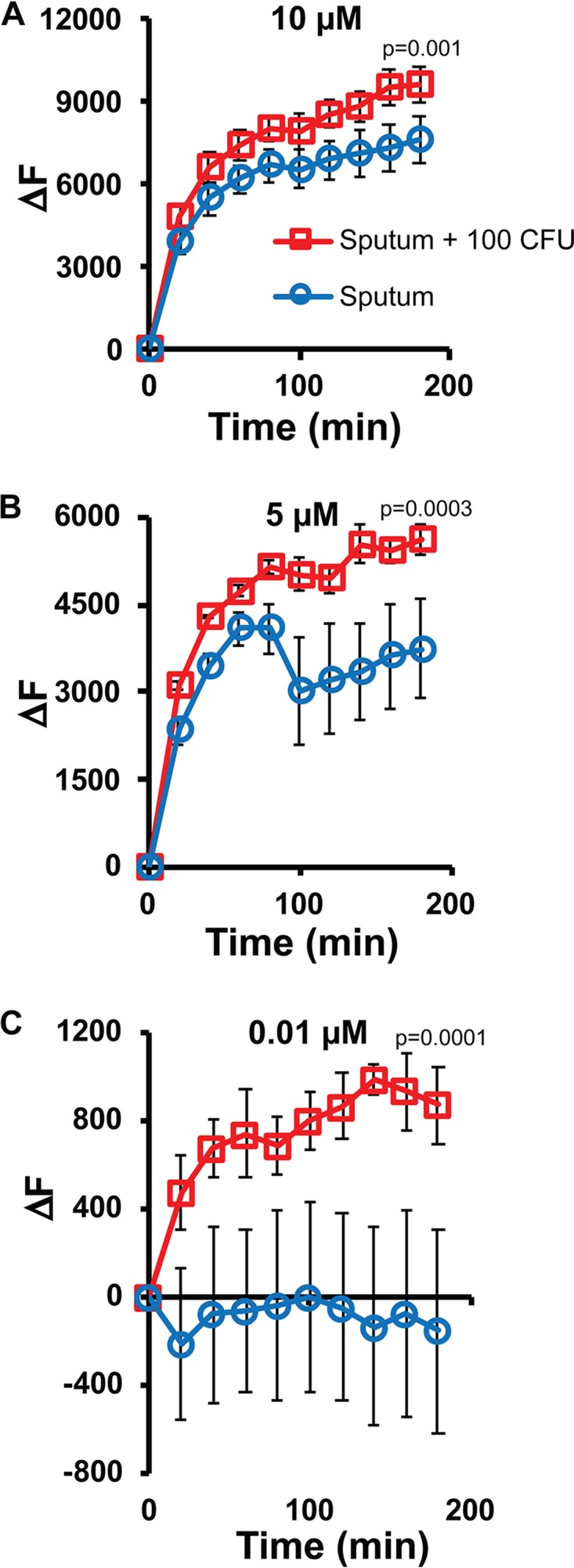
Detection of M. tuberculosis subsp. *bovis* bacillus Calmette-Guérin (BCG) in sputum using reporter enzyme fluorescence (REF) assays with the fluorogenic substrate CDG-3. CDG-3 at 10 μM (A), 5 μM (B), and 0.01 μM (C) was coincubated in sputum with transport stabilization solution (TSS) that contained 100 CFU of BCG. Every 20 min for 180 min at room temperature (25°C), the change in fluorescence (ΔF) was measured and calculated as the fluorescent signal (excitation, 490 nm; emission, 515 nm) at each time point minus the signal immediately after adding the substrate. *P* values shown on each line are derived from ANOVA comparison of data for sputum with 100 CFU BCG and sputum alone.

Culture can detect approximately 10 to 100 CFU of M. tuberculosis in sputum (70), making it important to optimize REF near this threshold. We have previously shown that tuberculosis-negative human sputum has large numbers of β-lactamase-producing bacteria (48, 49), making it necessary to validate the performance of any detection strategy directly in human sputum rather than laboratory medium. We were particularly interested in using assays at ambient temperatures, since a diagnostic strategy that does not require temperature control would be advantageous for low resource settings where tuberculosis diagnosis is most needed (52). REF assays detect 100 CFU of BCG, which expresses similar levels of BlaC to those of other M. tuberculosis strains (28, 40, 49, 50), spiked into human sputum within 20 min (Fig. 6). Interestingly, low concentrations of CDG-3 (0.01 μM) display improved signal to noise ratios, allowing use of very little substrate for assays. We selected 1 μM as the concentration for use in REFtb assays to ensure sufficient substrate is available in the complex environment present in clinical material where substrate may not always be readily accessible to the enzyme in solution. The sensitivity of these assays supports the feasibility of REF as a diagnostic assay for detection of tuberculosis infection using clinical material and suggests that its sensitivity may be similar to or better than that of culture.

### Evaluation of REFtb for diagnosis of tuberculosis in patients.

**FIG 7:**
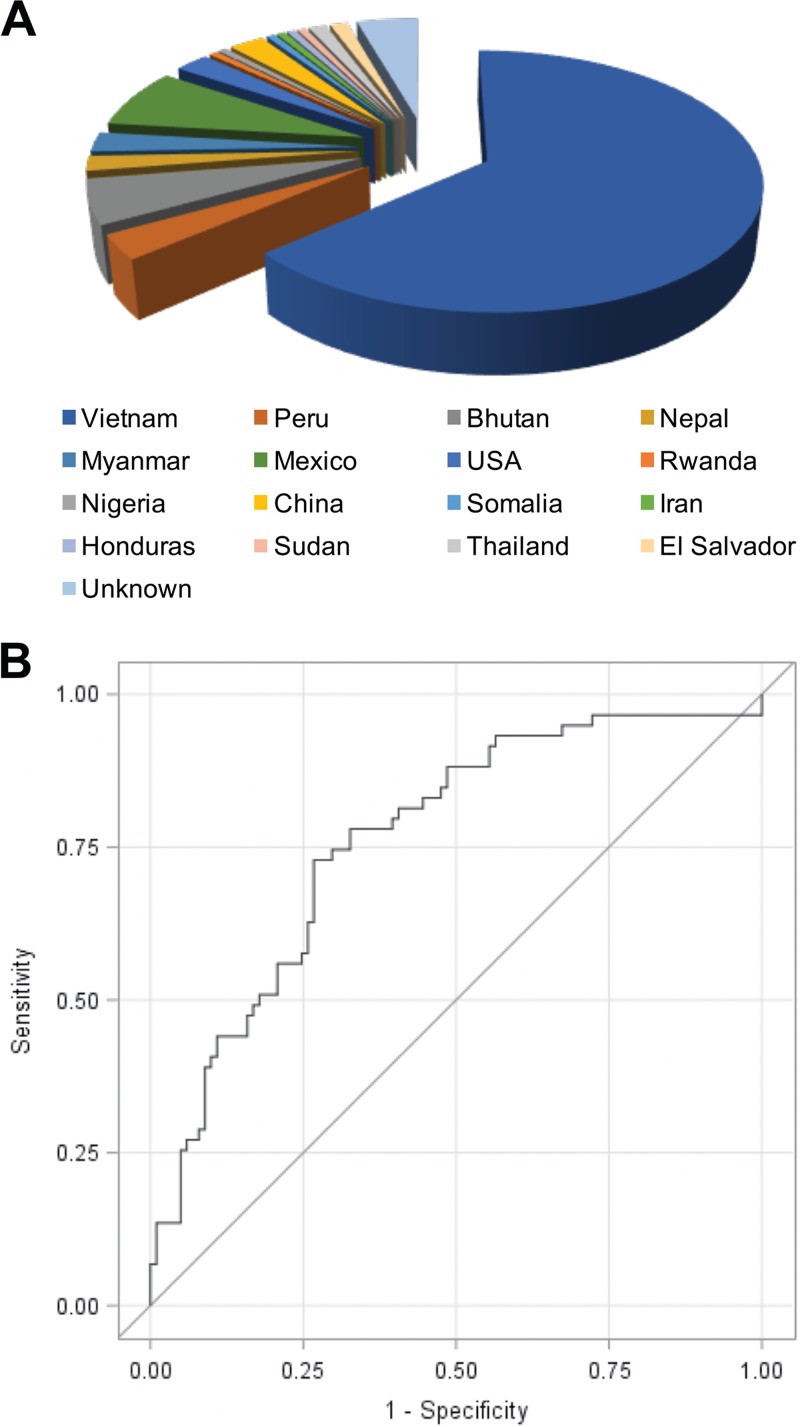
Country of origin and performance for clinical samples evaluated in the current study using the reporter enzyme fluorescence (REF) diagnostic assay. (A) Pie chart showing the percentage of total suspected tuberculosis cases analyzed using REF to identify tuberculosis (REFtb) from each country. Unknown indicates that no data are available for country of origin for those samples. The majority of samples were obtained from Vietnam (102, 64%), with Mexico (12, 8%) second and Bhutan third (9, 6%). (B) Receiver operating characteristic (ROC) curve analyzing all 160 samples for specificity and sensitivity of REFtb diagnosis against smear and culture diagnosis. The ROC curve was generated by plotting the sensitivity versus 1 − specificity using different cutoff points, giving an area under the curve of 76.27% (*P* = 0.0002).

We obtained 160 sputum samples from suspected tuberculosis patients, coded and blinded to evaluate the performance of REFtb as a diagnostic test for tuberculosis. Sputum samples were obtained from a geographically diverse population, from 16 countries, including the United States. (Fig. 7A). When we used a cutoff value threshold for the highest frequency of correct predictions of tuberculosis diagnosis, the REFtb assay yielded an overall sensitivity of 89% for smear- and culture-positive samples and 88% for smear-negative and culture-positive samples (Table 2), with a specificity of 82%. We analyzed REFtb diagnosis against smear and culture combined and applied receiver operating characteristic (ROC) curve analyses. ROC for all samples gives an area under the curve of 76.27% (Fig. 7B), demonstrating significant (*P* = 0.0002) correct predictive ability for REFtb diagnosis. The study population was 37.5% female, and no significant differences were observed for REFtb with respect to gender. REFtb performed similarly for all age groups for sensitivity (Table 2). However, specificity was slightly higher for patients <35 years old and >65 years old than for patients 36 to 65 years old (Table 2; *P* < 0.05). Clinical samples were assayed in 10 different batches of 7 to 20 samples each over 4 months. We found that there was variability in the signal obtained between batches for all samples in a batch. Thus, we explored the possibility that batch-specific analysis using the tuberculosis-negative sputum internal control in each batch for determining the cutoff threshold would be the best strategy for analysis of our data. When batch-specific analyses using the internal control from each batch are used, we obtain a sensitivity of 88.1% (95% CI = 77.1 to 95.1) and a specificity of 86.1% (95% CI = 77.8 to 92.2). The positive predictive value is 79% (*P* < 0.0001), and the negative predictive value is 93% (*P* < 0.0001). These data demonstrate that REFtb can diagnose tuberculosis with high sensitivity, and batch-to-batch variability can be corrected through batch-specific analysis using tuberculosis-negative sputum controls.

**TABLE 2:** Sensitivity and specificity of the reporter enzyme fluorescence diagnostic assay

Group (no. of samples)^*a*^	% Female (no.)^*b*^	S+, C+ (no.)^*c*^	S−, C+ (no.)^*d*^	Specificity (no.)^*e*^
Age range				
15–25 (11)	18.1 (2)	0 (1)	0 (0)	100 (10)
26–35 (33)	51.5 (17)	57.1 (7)	87.5 (8)	89 (18)
36–45 (38)	31.6 (12)	100 (11)	80 (5)	68 (22)^*f*^
46–55 (26)	19.2 (5)	100 (6)	66.6 (3)	76 (17)^*g*^
56–65 (37)	40.5 (15)	100 (7)	100 (6)	75 (24)^*h*^
>65 (15)	60.0 (9)	100 (3)	100 (2)	100 (10)
Sex				
Male (100)	0.0 (100)	92 (25)	93.3 (15)	82 (60)
Female (60)	100 (60)	80 (10)	77.7 (9)	81 (41)
Total no. (160)	37.5 (60)	89 (35)	87.5 (24)	82 (101)

a Clinical samples were divided into groups by age and sex to evaluate whether any of these differences impact the performance of the tuberculosis reporter enzyme fluorescent (REFtb) diagnostic assay. The total number of samples (n) in each group is shown in parentheses.

b The percentage of female patients within the group. The number of samples in this group that were from female patients is shown in parentheses.

c Sensitivity of the REFtb assay versus smear-positive (S+) and culture-positive (C+) samples calculated as the number of positives by REFtb divided by the number of positives by both smear and culture times 100 to make a percentage. The number (no.) of samples in this group that were smear positive and culture positive is shown in parentheses.

d Sensitivity of the REFtb assay versus smear-negative (S−) and culture-positive samples calculated as the number of positives by REFtb divided by the number of positives by culture only times 100 to make a percentage. The number of samples in this group that were smear negative and culture positive is shown in parentheses.

e Specificity of the REFtb assay-negative samples versus samples negative by both smear and culture calculated as the number of negatives by REFtb divided by the number of negatives by both smear and culture times 100 to make a percentage. The number of samples in this group that were both smear and culture negative is shown in parentheses. *P* values calculated using Boschloo’s exact test compared to the specificity of the >65 age group.

f *P* = 0.02.

g *P* = 0.06.

h *P* = 0.05.

## DISCUSSION

We report evaluation of BlaC as a biomarker that is conserved within the tuberculosis complex for rapid diagnosis. The unique characteristics of the BlaC active site, constitutive expression, and conservation in the tuberculosis complex make it an excellent biomarker (48–50). The REFtb assay requires no processing and can be accomplished in 10 min. We show that the CDG-3 probe represents a substantial improvement over other probes in that it is stable and shows negligible cross-reactivity with other bacteria. Very small amounts of CDG-3 can be used in these assays, making REFtb a very inexpensive test, expected to cost less than a dollar per sample. Affordability and simplicity make REFtb a promising option for POC use that would allow more TB cases to be identified. Examination of 160 clinical specimens from suspected tuberculosis-infected patients yielded a high sensitivity and specificity of 88.1% and 86.1%, respectively, obtained from ROC analyses with high statistical significance (*P* = 0.0002) and correct predictive value. These observations suggest REFtb is more sensitive than smear microscopy, which has a sensitivity of 20 to 80% (4). The negative predictive value of REFtb is 93% (*P* < 0.0001), suggesting that few cases are missed and emphasizing its potential for use as a rapid triage test. These values for specificity and sensitivity are directly in line with recommendations from the WHO for a biomarker-based triage test to identify suspected TB patients (71). We are particularly excited by the observation that REFtb can detect the majority of smear-negative cases that would be missed in diagnosis prior to obtaining culture results, since about 17% of tuberculosis transmission is thought to occur from these patients (72). Implementation of REFtb at the POC has the potential to facilitate early diagnosis of tuberculosis, potentially reducing diagnostic costs by 34 to 43% in the developing world (73).

REFtb fills a unique biological niche in diagnostic assays since it is a catalytic enzyme-based assay. Since BlaC is secreted by the Tat pathway that requires ATP (60), REFtb is amenable to evaluating therapeutic outcomes and phenotypic drug susceptibility tests (DST). Existing diagnostic strategies and most others in development are based on different biological mechanisms (52), either nucleotides (GeneXpert), cell wall staining characteristics (smear microscopy), bacterial replication (culture), the host response (QuantiFeron-TB), or antibody-based recognition of molecules produced by M. tuberculosis. These differences in the biological mechanisms make it likely that REFtb will serve as a diagnostic strategy that is complementary to others. While data obtained in this initial evaluation of REFtb for diagnosis of tuberculosis in clinical samples are promising, it is still early in development. A larger number of clinical samples from diverse regions should be examined to confirm the REFtb sensitivity and specificity, though this study produced high significance values (*P* = 0.0002). Optimally, batch variability should be eliminated to simplify analysis and prevent the need for inclusion of control samples. Batch variation is likely from preparation of the reagents, which currently are prepared fresh as liquids. Liquid reagents are not optimal for POC assays in limited resource settings. Stable lyophilized reagents are in development for REFtb that are expected to perform consistently from batch to batch, allow stable shipping at ambient temperatures, and eliminate solution preparation, facilitating use as a POC diagnostic assay.

Although the specificity of REFtb is very reasonable, particularly for a triage test in early development, some false-positives were obtained as defined by being negative by both smear and culture. There were no significant differences in the sensitivity between the age groups examined in this study, but there are small differences in specificity from the 36- to 65-year-old group that point toward a factor that can increase false positives. One possibility is that since REFtb is very sensitive, able to detect 1 to 10 bacteria in sputum (48–50), it is possible that some “false positives” are actually “true positives” that are missed by other tests and only identified by REFtb. Currently, we cannot differentiate true positives from true false positives, though follow up would allow us to differentiate them. With the sensitivity of smear microscopy at 20 to 80% (4) and most likely 50 to 60% (18, 74) and a single culture providing a sensitivity of ∼80% (15, 18), true positives can be missed by existing diagnostic strategies. We will conduct follow up in future studies that should allow us to differentiate true positives from false positives, but in the meantime, we are investigating other reasons.

Another possible explanation for false positives is that an unknown β-lactamase has a similar active site to BlaC or at least a similar ability to cleave CDG-3. We are investigating this hypothesis and, once a cross-reactive β-lactamase(s) is identified, incorporation of a specific inhibitor from the multitude of β-lactamase inhibitors available (75) and/or a specific antimicrobial that kills the cross-reacting bacterial species could possibly be used to eliminate these false positives and further improve REFtb specificity. Interestingly, the sensitivity of REFtb compared to both smear-positive and smear-negative tuberculosis samples is similar. This is most likely due to our choosing the most sensitive conditions possible for REFtb assays, where we read the samples after cleavage of the fluorogenic substrate plateaus, rather than while it is increasing linearly and remains quantitative. We previously observed this phenomenon with REF assays *in vivo*, where early time points allow quantitative determination of M. tuberculosis, but at later time points a similarly high signal is observed regardless of bacterial numbers present (28). Similarly, use of earlier time points in REFtb assays would likely allow quantitative measurement of bacterial numbers in sputum but would also most likely decrease sensitivity. Since we envision this test as a rapid triage test for tuberculosis, quantitation is less important than sensitivity, so we plan to retain the current assay conditions and focus on ensuring maximal sensitivity. Although false negatives are not common, we plan to investigate potential reasons that these occur to further improve the REFtb assay. One possible reason that samples that are positive by culture could become negative is improper storage and/or transport. This issue can be overcome by ensuring careful handling or by simply conducting the REFtb assays at the POC where bacterial viability is maintained. Since REFtb assays measure viable bacteria, we expect REFtb to perform even better on fresh samples than on samples that have been stored and shipped, even though the promising results from the current study were obtained using shipped samples. We envision REFtb as a triage test to be employed at the POC when a patient first visits a clinic. Management after identification may ultimately involve treatment or careful monitoring in parallel with additional testing, but with a negative predictive value of 93%, REFtb could be used to focus resources on those patients most likely to have tuberculosis.
